# Presence of Microorganisms in the Environment: One Health Approach

**DOI:** 10.3390/microorganisms13112435

**Published:** 2025-10-23

**Authors:** Helen Haydee Fernanda Ramirez-Plascencia, Ana Gabriela Colima-Fausto, Karel Cesar Licona-Lasteros, Mariana Díaz-Zaragoza, Gerardo Cazarez-Navarro, Jose Guadalupe Macias-Barragan, Sergio Yair Rodriguez-Preciado

**Affiliations:** 1 Unidad Academica de Ciencias de la Salud, Departamento Académico, Aparatos y Sistemas 2, Universidad Autónoma de Guadalajara, Avenida Patria 1201, Zapopan 45129, Jalisco, Mexico; helen.ramirez@edu.uag.mx; 2 Departamento de Ciencias Basicas Medicas, Escuela de Medicina y Ciencias de la Salud, Tecnologico de Monterrey Campus Guadalajara, Avenida General Ramón Corona No 2514, Zapopan 45201, Jalisco, Mexico; 3 Laboratorio de Sistemas Biológicos, Departamento de Ciencias de la Salud, Centro Universitario de los Valles, Universidad de Guadalajara, Carretera Guadalajara—Ameca 45.5Km, Ameca 46600, Jalisco, Mexico; karel.licona@academicos.udg.mx (K.C.L.-L.); mariana.diaz@academicos.udg.mx (M.D.-Z.); josemacias@valles.udg.mx (J.G.M.-B.); 4 Secretaria de Salud Jalisco, Zona Centro, Dr. Baeza Alzaga 107, Guadalajara 44100, Jalisco, Mexico; gecana55@hotmail.com

**Keywords:** one health, antimicrobial, bacteria, virus, fungi, parasites, environment, zoonoses

## Abstract

The One Health approach offers an integrative framework to understand infectious threats, environmental factors, antimicrobial resistance (AMR) and how their interactions affect the human–animal–environment interface. This review examines the epidemiology, transmission pathways, and mechanisms of microorganisms of public health importance (bacteria, fungi, parasites, and viruses). It highlights the interconnectedness of ecosystems, where the environment plays a central role in the dissemination of pathogens, driven by climate change, globalization, agricultural intensification, and habitat degradation. AMR is a major concern, driven by the indiscriminate use of pharmaceuticals in human, veterinary, and agricultural settings, horizontal gene transfer through mobile genetic elements, and microbial evolution. The study of different pathogens is of great importance due to their high prevalence in different ecosystems, their virulence, clinical interest, and mortality rates produced. Some of them are ESKAPE bacteria, *Candida auris*, *Plasmodium falciparum*, and emerging viruses such as SARS-CoV-2, which present complex transmission dynamics influenced by ecological and health determinants. The review also addresses the effects of climate change on the persistence and geographic spread of pathogens. Successful implementation of the One Health program requires intersectoral policies, integrated surveillance systems, prudent use of antimicrobials and investment in translational science. Coordinating these strategies is essential to limit the spread of pathogens, protect biodiversity, and save global health in the face of the growing threat of infectious diseases.

## 1. Introduction

One Health is an emerging concept that seeks to sustainably improve the well-being of humans, animals, and ecosystems [1]; addressing current and emerging health threats at the human–animal–environment interface [2]. The importance of One Health has increased due to the rising incidence of infectious diseases, many of which are of environmental zoonotic origin. Around 60% of human infections have an animal origin, of which approximately 75% are considered emerging or re-emerging diseases. The remaining 40% co-evolved with humans or arose from non-zoonotic environmental sources [3,4].

Furthermore, the environment acts as a reservoir and transmission route for pathogens [5]. This, coupled with scientific evidence showing a relationship between climate change and antimicrobials, has led to altered microbial ecosystems and enhanced resistance mechanisms. Different environmental conditions have been reported to favor the rise in antimicrobial resistance (AMR). An increase of ten degrees Celsius in ambient temperature has been linked to a four to five percent increase in resistance prevalence [6]. Water scarcity and flooding promote the development of antibiotic-resistant bacteria by contaminating drinking water with antibiotic residues and other bacteria [7]. Prolonged droughts increase reliance on unsafe water sources, increasing exposure to pathogens [8].

Furthermore, AMR has become a global concern due to the overuse of antibiotics; 30% of antibiotics are used for human consumption and 70% are used in livestock [9]. The excessive intake and misuse of antimicrobials, as well as sources of contamination such as wastewater from pharmaceutical industries and hospitals due to poor treatment, agricultural runoff, and improper disposal of unused or waste drugs, exert a selective pressure that favors the survival and proliferation of resistant strains [9,10,11,12]. These events have accelerated the emergence of mutations and genetic variants that modify the transcriptional response to drug administration, leading to natural evolutionary mechanisms, as well as the presence of mobile genetic elements that facilitate horizontal gene transfer. In combination, these processes allow microorganisms to acquire resistance to multiple classes of antimicrobials [13,14,15]. Therefore, the aim of this review is to analyze the presence and dissemination of microorganisms, as well as antimicrobial resistance, from a One Health perspective, in order to highlight their impact on global public health.

## 2. Bacteria

### 2.1. Zoonotic Bacterial Infections

Zoonotic bacterial infections represent an increasing threat to global public health, particularly in contexts where humans are in close contact with domestic and wild animals [16,17]. Over 60% of known human pathogens are of zoonotic origin [18], including bacteria transmitted via direct contact, contaminated food, vectors, or environmental exposure [19,20,21]. For example, *Rattus norvegicus* serves as a major reservoir in urban settings, harboring bacterial pathogens such as *Leptospira interrogans* with prevalences between 1% and 83%, *Bartonella* spp. between 17% and 38.6%, *Escherichia coli* between 46% and 77%, *Salmonella* spp. between 0.5% and 3.6%, *Borrelia* spp. up to 42.9%, *Rickettsia* spp. between 0.8% and 19.5%, *Campylobacter jejuni* with 4%, *Clostridium* difficile between 1% and 39.2%, *Coxiella burnetii* between 1.3% and 8.7%, and *Streptobacillus moniliformis* up to 23%. Likewise, other *Enterobacteriaceae* have been identified, such as *Klebsiella* spp. (13%), *Enterobacter* spp. (7%), *Serratia* spp. (2%), *Proteus* spp. (2%), *Shigella* spp. (1%), and *Citrobacter* spp. (1%), as well as methicillin-resistant *Staphylococcus aureus* (3.5–3.7%) [22,23]. The transmission routes of these pathogens through *Rattus novergicus* may be by direct contact, bites and scratches, exposure to hematophagous arthropods (fleas, ticks, lice, and mites), and ingestion of water or food contaminated with urine or feces. Furthermore, since the pathogens are eliminated through urine, saliva, and feces, they result in contamination of surfaces, water, and food sources [17,24,25].

The complexity of the human–animal interface is driven by factors such as rapid urbanization, agricultural intensification, global trade in animals and animal products, and the rising trend of pet ownership [26]. These conditions heighten the risk of zoonotic exposure and have contributed to the emergence of over 250 zoonotic diseases in the past 70 years [27]. Notably, bacterial species such as *Campylobacter* spp. and *Salmonella* spp., frequently found in livestock and pets, are implicated in numerous outbreaks of human disease [28,29,30,31]. In Europe, zoonotic bacterial transmission has been linked to multiple routes including ingestion of contaminated food, inhalation of aerosolized particles, consumption of raw or undercooked animal products, and contact with contaminated environments [32].

Importantly, zoonotic transmission is not exclusively animal-to-human. Cases of reverse zoonosis, where pathogens are transmitted from humans to animals, have been increasing [33]. Some reports, such as the transmission of *Mycobacterium tuberculosis* and *M. bovis* from farmers to cattle in Nigeria and Ethiopia, from caretakers to elephants in India, and of *M. orygis* from humans to antelope and deer in Chennai, have emerged. Methicillin-resistant *Staphylococcus aureus* has been transmitted from farmworkers to cattle and pigs in Australia, Germany, the United States, and Taiwan, while extended-spectrum Beta-lactamase (ESB)-producing *Escherichia coli* strains have been detected in dogs and horses in Europe. *Salmonella enteritidis* has also been reported in seabirds and poultry in remote regions such as Antarctica. *Helicobacter pylori* infections have been reported in captive marsupials in Australia and *Campylobacter* spp. in gorillas in Uganda, further highlighting the human–animal connection [34].

The emergence of highly virulent hybrid strains underscores the public health risk of such interactions. A notable case is the 2011 outbreak of *Escherichia coli* O104:H4 in Europe, attributed to genetic recombination between human- and animal-associated strains [34]. This recombination process is suspected to have occurred in the environment, on a farm, where the bacteria would have been exposed to the phage and plasmid in the soil or on seeds, leading to the development of the O104:H4 strain [35]. which resulted in numerous cases of hemolytic uremic syndrome and fatalities [36]. This event highlights the critical need to adopt a One Health approach, acknowledging the interconnection between human, animal, and environmental health.

### 2.2. Effect of the Environment on Bacteria Infections

The environment constitutes a dynamic reservoir for pathogenic bacteria, many of which pose a significant threat to human health [37], especially in settings characterized by fecal contamination, anthropogenic activity, and inadequate sanitation infrastructure [38,39,40]. Pathogenic bacteria have been reported in diverse environmental matrices including water, air, soil, food, and built environments such as hospitals, HVAC systems, and medical equipment [41,42,43]. This widespread presence is largely due to bacterial resilience mechanisms, such as biofilm formation and resistance to disinfectants, enabling survival under harsh conditions [43,44,45].

Airborne bacterial communities, including genera such as *Mycobacterium*, *Coxiella*, *Legionella*, *Chlamydophila*, *Staphylococcus*, and *Streptococcus*, have been detected in bioaerosols, many of which are associated with respiratory infections [46]. The detection of viable pathogens in air suggests their capacity to survive under extreme conditions and disperse through wind or precipitation, presenting a potential route for human exposure and disease transmission [47].

In freshwater ecosystems subject to anthropogenic influence, contamination by wastewater, agricultural runoff, and diffuse fecal pollution introduces diverse bacterial pathogens. Reported genera include *Salmonella*, *Escherichia*, *Shigella*, *Yersinia*, *Klebsiella*, *Leptospira*, *Vibrio cholerae*, *Aeromonas hydrophila*, *Legionella pneumophila*, *Mycobacterium*, and *Pseudomonas*, among others [40,48,49,50].

Drinking water distribution systems also serve as colonization sites for bacteria such as *Legionella* spp., *Mycobacterium avium*, and *Pseudomonas aeruginosa*, particularly in the presence of biofilms in pipes, storage tanks, faucets, and cooling towers [43,48,51]. In a study by Makovcová et al. in 2014 [51], they conducted their study in various freshwater environments located in the Czech Republic, including fish ponds, drinking water reservoirs, storage ponds, and an experimental recirculation system. They reported non-tuberculous mycobacteria (NTM), detected in 23.7% of 396 water samples from ponds, reservoirs, and recirculating systems. The identified species—*Mycobacterium asiaticum*, *M. chimaera*, *M. interjectum*, and *M. simiae*—were associated with pulmonary infections in humans and reported for the first time in freshwater bodies, representing a novel environmental health risk [51].

ESKAPE pathogens are of particular concern due to their multidrug resistance and high virulence, especially in healthcare settings [52,53,54]. Waste water and hospitals act as important reservoirs for these bacteria, contributing to healthcare-associated infections [54,55]. Furthermore, various environmental studies report that this group of bacteria exhibits a wide distribution and significant resistance profiles in different reservoirs. *Enterococcus faecium* has been isolated from livestock in Zambia with resistance to gentamicin (96.6%), cotrimoxazole (89.7%), and penicillin (79.3%); in wastewater and receiving waters in Portugal, it showed resistance to linezolid (41%) and tetracycline (40%); in soils and waters in Brazil, it was detected with resistance to fluoroquinolones (100%) and erythromycin (92.5%). *Staphylococcus aureus* was identified on public beaches with resistance to methicillin (60%); in beach sand and water, it showed resistance greater than 70% to penicillin, rifampicin, and clindamycin; in treated wastewater in South Africa, it presented high levels of resistance to β-lactams and lincosamides. *Klebsiella pneumoniae* was isolated from European rivers and lakes with varying resistance levels (neomycin 50.9%, tetracycline 9.1%); in Croatia, strains resistant to carbapenems and cephalosporins were identified in 100% of isolates. *Acinetobacter baumannii* was detected in the Seine River and European landfills with multidrug resistance. Regarding *Pseudomonas aeruginosa*, isolates from European wastewater and rivers were documented, with multidrug-resistant strains (7.1%) and resistance to aztreonam (70.4%) in Poland. Finally, *Enterobacter* species were reported in the Narmada River (India), with *blaTEM* (42%) and *blaSHV* (44%) genes [56].

The interplay between environmental contamination (water, air, soil, and surfaces) and human susceptibility factors—such as immunosuppression, poverty, or malnutrition—creates a cycle of exposure and infection. Thus, integrated strategies are needed, including robust environmental microbiological surveillance (targeting both traditional indicators and emerging pathogens), improved sanitation measures, and preventive interventions to mitigate the burden of environmentally associated bacterial infections [57].

**Figure 1:**
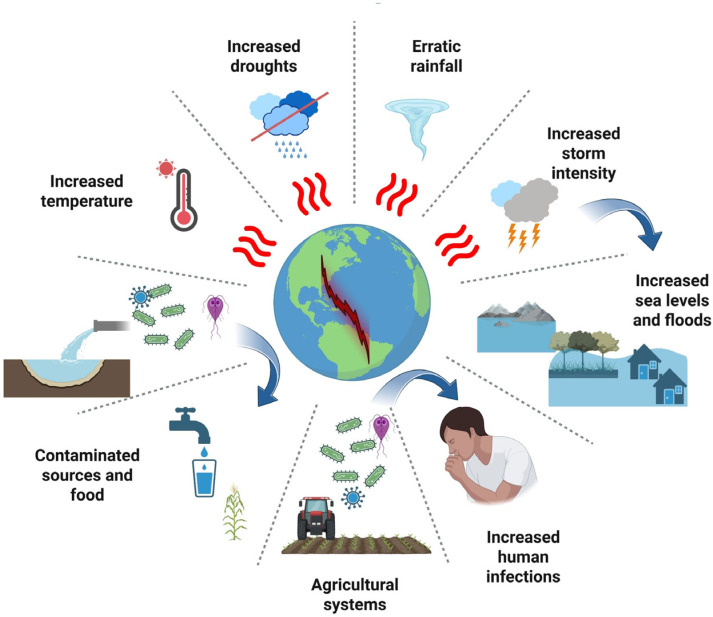
Pathogen movement from floodwaters to human exposure.

Climate change is a key driver of bacterial persistence, dispersal, and pathogenicity in environmental settings. Altered temperature, humidity, and precipitation patterns reshape both natural and anthropogenic ecosystems, creating favorable conditions for the survival and transmission of bacterial pathogens [58,59]. Extreme weather events such as flooding, droughts, or heavy rainfall can mobilize pathogens from manure, wastewater, and contaminated soils into drinking water sources and agricultural systems, compromising water quality and increasing the incidence of waterborne infections, particularly in vulnerable regions (Figure 1) [60,61,62,63].

Globally, rising temperatures and precipitation have been linked to increased environmental loads of human-pathogenic bacteria such as *Escherichia coli*, *Klebsiella pneumoniae*, *Salmonella* spp., and *Listeria monocytogenes* [64]. Warm and humid conditions enhance the persistence of pathogens like *Salmonella* spp., *Listeria monocytogenes*, and *Campylobacter* spp., promoting their dissemination through agricultural production systems, food-processing surfaces, and the food supply chain [65].

Moreover, bacteria harboring multiple AMR genes exhibit a greater ecological versatility, suggesting that climate change may also contribute to the global spread of multidrug-resistant bacteria [64].

### 2.3. Antibiotic Resistance

Antibiotic resistance (AR) is a multifactorial outcome of accelerated bacterial evolution driven by human activities and represents a global threat that extends well beyond clinical settings. AR and the One Health paradigm are inherently interconnected, as the emergence and spread of resistant bacteria are governed by the interplay among human, animal, and environmental health. This framework recognizes that AR is not solely a human or animal health problem or confined to clinical contexts, but rather a complex challenge requiring coordinated action across all three sectors to achieve improved community health and well-being [66].

The One Health paradigm provides the optimal framework for understanding and addressing the multifaceted dimensions of antibiotic resistance, from the genetic selection of resistant bacteria to their intersectoral dissemination [67,68,69].

Indiscriminate antibiotic use, selective pressure, horizontal gene transfer, and the involvement of various biological and environmental vectors all significantly contribute to the emergence and dissemination of antibiotic-resistant bacterial strains [70]. Therefore, the concept of antibiotic resistance should not be limited to the bacteria themselves but should also encompass the repertoire of antibiotic resistance genes (the resistome), antibiotic resistant patients, and healthcare facilities exhibiting high resistance rates, food-producing animals, and environmental reservoirs such as water and soil [71]. By integrating and mapping these elements under the One Health framework, researchers can elucidate the dynamics of resistant infections and their transmission pathways ultimately guiding interventions to reduce antibiotic resistance and prevent its further spread. Recent initiatives such as large-scale resistome mapping studies and the MetaSUB project, which monitors antimicrobial resistance genes across urban environments worldwide, exemplify the potential of these approaches to guide interventions aimed at reducing antibiotic resistance and preventing its further spread [72,73,74].

The microbiomes associated with humans, animals, plants, aquatic environments, and soils form an interconnected network that allows, albeit to a limited extent, the exchange of the bacterial pangenome, including antibiotic resistance genes. Although membrane-based computational models have recently been developed to probe the complex dynamics of antibiotic resistance, there remains a critical need for quantitative studies that elucidate the specific contributions of each microbiome and its ecosystem to the emergence and dissemination of bacterial resistance [75,76].

In 2017, the World Health Organization (WHO) published a global priority list of antibiotic-resistant pathogens, which included carbapenem-resistant *Acinetobacter baumannii*, *Pseudomonas aeruginosa*, *Enterobacteriaceae* resistant to carbapenems or producing extended-spectrum β-lactamases, *Salmonella* spp., fluoroquinolone-resistant *Campylobacter* spp., vancomycin-resistant *Enterococcus faecium*, and methicillin-resistant or intermediate-resistant *Staphylococcus aureus*. In wildlife, antibiotic resistance research has predominantly focused on *Escherichia coli*, *Salmonella* spp., and *Enterococcus* spp., with fewer studies addressing *Campylobacter* spp. and *S. aureus* [77,78]. However, these studies are constrained by significant methodological limitations, such as small sample sizes, design biases, variability in detection techniques, and limited geographic and temporal coverage, which undermine the validity of their findings and necessitate cautious interpretation when comparing across species, bacterial groups, and regions [79].

Environmental compartments act as “resistance amplifiers” when contaminated by antibiotics or resistant bacteria. Hospital and municipal effluents carry high loads of ESBL-producers and carbapenemase-positive *Enterobacteria*. Advanced oxidation processes (AOPs) and membrane bioreactors can reduce AMR determinants by more than 90%, although their implementation is still limited in low- and middle-income countries. In Southeast Asia, chlorinated surface waters showed >60% of *E. coli* isolates resistant to ampicillin and trimethoprim-sulfamethoxazole, reflecting inadequate wastewater regulation and highlighting the risk of resistance dissemination at the human–environment in-terface. Agricultural soils fertilized with manure harbor a prevalence greater than 20% of tetracycline determinants (e.g., *tetM*, *tetW*), confirming the role of farming practices in shaping the environmental resistome [80,81,82,83]. Moreover ticks collected from livestock were shown to carry AMR determinants against aminoglycosides, β-lactams, fluoroquinolones and tetracyclines, demonstrating that arthropod microbiomes form an overlooked conduit for AMR transmission between animals and environment [84].

Antibiotic use in livestock drives selection before pathogens ever reach humans. Metaphylactic administration of ceftiofur in poultry has been shown to drive a dramatic increase in resistance, with the proportion of Salmonella Heidelberg isolates resistant to third-generation cephalosporins rising from approximately 5% to 35% over a single production cycle, a trend mirrored by parallel increases in human clinical cases [66,85]. These findings underscore the importance of enforcing strict withdrawal periods to prevent antimicrobial residues from entering the food chain, as well as the implementation of regulatory frameworks that limit the non-therapeutic use of critically important antimicrobials in food-producing animals [85]. Likewise, subtherapeutic dosing of florfenicol in calves not only enriched intestinal Escherichia coli populations harboring multidrug-resistance plasmids but also disrupted short-chain fatty acid profiles, thereby compromising animal health and creating reservoirs with zoonotic potential [86]. In plant agriculture, routine sprays of streptomycin and tetracycline on fruit orchards elevated the prevalence of streptomycin-resistant *Pseudomonas syringae* to over 40% within leaf-surface microbiomes, illustrating a feedback loop whereby agricultural use of antibiotics propagates resistance that can cycle back into animal and human populations [87].

In hospitals, multidrug-resistant organisms (MDROs) fuel severe infections. In Latin American intensive care units, carbapenem resistance among *Acinetobacter baumannii* and *Pseudomonas aeruginosa* routinely exceeds 50%, underscoring the intense selective pressure exerted by broad-spectrum β-lactam use [88]. Simultaneously, *Klebsiella pneumoniae* harboring the NDM-1 metallo-β-lactamase have been isolated both from bloodstream infections and from sink drains, revealing biofilm niches that withstand standard disinfection protocols [89]. Moreover, transmission pathways clearly extend beyond direct patient-to-patient spread to include contaminated surfaces, plumbing systems and even hospital-adjacent soil microbiota, highlighting the critical environmental dimensions of nosocomial antibiotic-resistance dissemination [90].

A harmonized surveillance system spanning human, veterinary and environmental domains, exemplified by the WHO’s GLASS initiative and complementary regional networks, enables coordinated tracking of resistance trends across sectors. Parallel to this, rigorous antimicrobial stewardship protocols must be enforced to restrict the use of critically important antibiotics in animal production and to optimize prescribing practices in human healthcare [66,91]. At the same time, deploying innovative wastewater treatment technologies, such as ultraviolet/ozone treatment and photocatalysis, can intercept antibiotic-resistant bacteria and AMR determinants before they are discharged into natural ecosystems [92]. Recent meta-analyses indicate that advanced oxidation processes (AOPs) can achieve 70–95% reductions in antibiotic resistance genes, with combined ozone/UV treatment and membrane bioreactors showing the highest effectiveness across diverse wastewater settings [93,94,95,96]. Finally, robust policy and education efforts, including regulatory bans on non-therapeutic antibiotic applications, crackdowns on substandard or falsified drugs, and cross-sector training programs, are essential to raise awareness of resistance risks and to underpin all One Health interventions.

Recent studies converge on the view that AR emerges and circulates through a tightly woven network spanning humans, animals and the environment. Quantitative estimates—from 1.7% *mcr-1* prevalence in wildlife to >60% resistance in contaminated waters—underscore the urgency of a One Health paradigm. Only by synchronizing surveillance, stewardship and infrastructure improvements across all sectors can we hope to curb the relentless spread of antibiotic resistance (Table 1).

## 3. Fungal

### 3.1. Fungal Infections

Fungal infections pose a significant global health threat, with an estimated 6.5 million invasive infections and 3.8 million related deaths each year [106]. It is estimated that invasive aspergillosis impacts over 2.1 million individuals annually, with an 85.2% mortality rate. At the same time, bloodstream infections produced by *Candida* lead to around 995,000 deaths each year, resulting in a mortality rate of 63.6% [106]. In 2022, the World Health Organization released its first Fungal Priority Pathogens List (WHO-FPPL), which categorized 19 fungal pathogens into three groups: critical (*Candida auris*, *C. albicans*, *Cryptococcus neoformans*, *Aspergillus fumigatus*), high (*Nakaseomyces glabratus*, *C. parapsilosis*, *C. tropicalis*, eumycetoma agents, *Fusarium* spp., *Mucorales*, and *Histoplasma* spp.), and moderate priority, based on unmet research and development needs and their public health importance [107,108].

The One Health framework emphasizes the connections between human, animal, and environmental health. It highlights that human health is closely linked to the health of animals and the environment we share [109]. This approach is particularly important for fungal infections, inasmuch about 75% of emerging infectious diseases emerged from animals. Many harmful fungi also have environmental reservoirs (including soil systems, aquatic environments, air masses, plant tissues, endophytic associations, and anthropogenic environments such as composting facilities and water treatment systems) that help them spread across species [110]. Using One Health principles for fungal diseases is crucial for understanding how diseases emerge, how they spread, the patterns of drug resistance, and how to develop effective prevention and control strategies.

### 3.2. Zoonotic Mycoses

Dermatophytoses are the most common fungal infections affecting both animals and humans. They have significant zoonotic potential that illustrates the One Health concept [111]. These infections are caused by fungi that thrive on keratin and are categorized by their ecological preferences: anthropophilic (human-adapted), zoophilic (animal-adapted), and geophilic (soil-dwelling) species. Despite their global impact and considerable illness, dermatophytes were not included in the WHO-FPPL due to the list’s explicit focus on invasive fungal diseases with high mortality rates. The WHO prioritization framework emphasized life-threatening systemic infections, hospital-acquired resistant pathogens, and conditions affecting severely immunocompromised patients, rather than superficial cutaneous mycoses that primarily impact quality of life [112]. This exclusion indicates a notable gap in recognizing the broader public health importance of dermatophytoses within the One Health framework.

Recent global surveillance data from 2009 to 2024 shows that cats, dogs, cattle, rabbits, rodents, hedgehogs, and horses are the most commonly reported animals infected with dermatophytes that can spread to humans [113]. *Microsporum canis*, mainly linked to cats and dogs, causes most pet-related dermatophytosis cases in humans, especially leading to tinea capitis with kerion development in children. A thorough retrospective study covering 15 years showed that *M. canis* remains the leading zoonotic dermatophyte worldwide, with prevalence rates between 23% and 67% in domestic animal populations, depending on the region [114]. *Trichophyton verrucosum* is strongly associated with cattle, while the *T. mentagrophytes* complex has a diverse range of hosts, with rabbits being frequently reported [113]. Recent data from veterinary clinics in Europe indicates that dermatophytosis affects about 2–5% of companion animals annually, with even higher rates in shelters, reaching up to 18% [115].

The epidemiological landscape of zoonotic dermatophytosis has changed significantly in recent years. *Trichophyton benhamiae*, typically associated with contact with infected guinea pigs, has become a major concern since its first report in Japan in 2002 and subsequent identification in Germany in 2011. In Germany, *T. benhamiae* has become the most commonly isolated zoophilic species in human infections, especially in children, with prevalence rates in guinea pig farms and pet shops ranging from 16.8% to 93.0%, and more than 90% of infected animals being asymptomatic carriers [112]. However, *T. benhamiae* presents significant diagnostic challenges due to morphological similarity to other dermatophyte species, requiring molecular confirmation for definitive identification. Internal transcribed spacer (ITS) sequencing remains the gold standard diagnostic test but faces limitations in distinguishing *T. benhamiae* from closely related species in the *T. mentagrophytes* complex, with specimens sometimes showing 98–99% identity to multiple species. Clinical diagnosis is further complicated by highly inflammatory presentations often misidentified as bacterial infections [116].

Likewise, *T. quinckeanum*, traditionally related to mouse favus, has turned into the third most frequently isolated zoophilic dermatophyte in some European regions. It was responsible for 19.0% of human zoonotic infections from 2014 to 2021 [112]. The COVID-19 pandemic has worsened these trends, leading to a notable rise in human infections caused by zoonotic dermatophytes. This increase may be due to the higher adoption of asymptomatic infected animals as pets, less access to veterinary care, and more human-animal contact [112].

### 3.3. Effect of the Environment in Fungal Infections

Anthropogenic climate change is modifying the epidemiology of fungal infections, affecting 58% of all known human infectious diseases [117]. Rising temperatures and changing precipitation patterns are impacting fungal growth, distribution, and virulence. This could contribute to the geographic spread of harmful fungi and increase human exposure to new, potentially more virulent or resistant strains [117].

Rising global temperatures are promoting thermotolerance, allowing them to survive and grow at mammalian body temperatures. This is illustrated by *Candida auris*, which is believed to have evolved from a plant saprophyte to a human pathogen after adapting to higher temperatures associated with climate change. The “thermal mismatch hypothesis” suggests that the mammalian immune system’s reliance on fever to fight infections might become less effective as fungi adapt to warmer temperatures [117].

**Figure 2:**
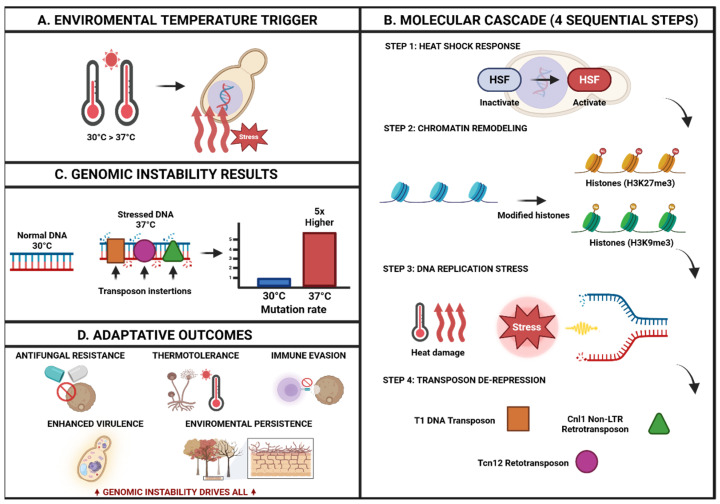
Heat stress-induced transposon mobility drives genomic instability and adaptation in pathogenic fungi. (**A**) An increase in environmental temperature from 30 °C to 37 °C triggers a cellular stress response in *Cryptococcus neoformans*. (**B**) Four-step molecular cascade: (1) Heat shock factor (HSF) activation, (2) Chromatin remodeling with histone modifications (H3K27me3, H3K9me3), (3) DNA replication stress with damage accumulation, (4) De-repression of three transposon types (T1 DNA transposon, Tcn12 retrotransposon, Cnl1 non-LTR retrotransposon). (**C**) Resulting 5-fold increase in mutation rate with widespread transposon insertions creating genomic instability. (**D**) Adaptive outcomes including antifungal resistance, thermotolerance, immune system evasion, enhanced virulence, and environmental persistence. Based on Gusa, A., et al. [118].

Recent experimental findings show that heat stress increases transposon mobility in pathogenic fungi like *Cryptococcus deneoformans*, leading to widespread genetic instability and potential adaptation strategies. Landmark research revealed a genome-wide, 5-fold increase in transposon mutation rates at 37 °C versus 30 °C, with three distinct transposable elements (T1 DNA transposon, Tcn12 retrotransposon, and Cnl1 non-LTR retrotransposon) showing temperature-dependent mobilization. This mechanistic process follows a stress response cascade: temperature stress triggers heat shock factor activation, leading to chromatin remodeling, DNA replication stress, transposon de-repression, and ultimately genomic rearrangements that facilitate rapid adaptation to environmental and host defense changes (Figure 2) [118].

Climate change has led to the spread of several endemic fungi beyond their usual geographic areas. For instance, *Coccidioides* species, which cause Valley fever, exemplify this change. Recent data from the Centers for Disease Control and Prevention (2019) revealed substantial increases in coccidioidomycosis in areas where it was not previously common, with rates reaching 187.8 cases per 100,000 in Arizona and expanding into regions that were previously unaffected [119].

Similarly, *Cryptococcus gattii*, which historically thrived in tropical and subtropical areas, has now established itself in temperate zones, including the Pacific Northwest of North America. A thorough analysis of its geographic distribution has confirmed the spread of the *C. gattii* complex into Europe, with climate models forecasting further northward movement due to rising temperatures [120]. The distribution of dimorphic mycoses in the United States has fundamentally shifted. *Histoplasma capsulatum* is now found in regions once thought to be non-endemic, challenging old beliefs about exposure risk [121].

### 3.4. Surveillance and Diagnostic Challenges in One Health Implementation

Effective One Health strategies for monitoring fungal diseases need integrated tracking across human, animal, and environmental sectors. Currently, surveillance systems are often disjointed, with limited coordination among medical, veterinary, and environmental health authorities. The WHO-FPPL has underscored the need for a better surveillance infrastructure, especially in resource-limited regions where the burden of fungal disease is the highest but the capacity for diagnosis is the lowest [108].

Diagnosis of fungal diseases remains difficult across all sectors. Many fungal infections lack rapid, sensitive, and affordable tests, leading to diagnostic delays and inadequate treatment. This lack of diagnosis increases the burden of disease and increases drug resistance due to the empirical use of antifungals.

Recent advancements in molecular diagnostics have transformed fungal detection. Next-generation sequencing methods show better performance showing 95–99% sensitivity and specificity compared to traditional culture techniques [122]. New nucleic acid-based diagnostic platforms can deliver results in less than 5 h, compared to 14 days with standard techniques [123]. PCR-based systems and rapid antigen detection tools are particularly promising for use in both clinical and field settings, with several achieving sensitivity rates above 90% for major fungal pathogens [124].

### 3.5. Antifungal Resistant

The rise of *Trichophyton indotineae* is an example of how anthropophilic dermatophyte clones can emerge and spread globally, often showing significant resistance [112]. This species causes widespread, chronic, and challenging dermatophytosis on the Indian subcontinent and has shown resistance rates of up to 75% to terbinafine and 25% to itraconazole in certain cases. Since then, it has spread worldwide, with cases reported on every continent, raising concerns about local circulation outside its original area [112].

Molecular analysis indicates that terbinafine resistance in dermatophytes mainly stems from point mutations in the *SQLE* gene, which encodes squalene epoxidase. The most common substitutions are p.Phe397Leu and p.Leu393Phe [125]. These mutations result in considerable increases in minimum inhibitory concentrations to terbinafine, with resistance rates reaching up to 4.3% for *T. interdigitale* and 3.6% for *T. rubrum* in recent surveillance data from the United States [126]. Recent Canadian surveillance (2014–2023) documented the emergence of terbinafine-resistant *T. indotineae* with *SQLE* mutations, confirming the global spread of this resistant clone [127].

The rise of antifungal resistance is one of the pressing challenges of the One Health framework in medical mycology. Azole compounds, especially triazoles, are widely used in human medicine, veterinary practice, and agriculture. As a result, resistance is evolving independently in different environments, which has serious implications for human health.

The use of agricultural fungicides is directly linked to the rise of triazole-resistant *Aspergillus fumigatus* infections in patients without prior antifungal exposure. Recent plant microbiome studies demonstrate that foliar fungicides significantly reduce abundance of beneficial fungal communities. Noel et al. (2022) showed that 238 unique fungal operational taxonomic units were differentially affected in soybean phyllosphere, and network complexity decreased significantly following fungicide treatment [128]. Analysis of triazole fungicide use in the United States from 1992 to 2016 revealed a significant four-fold increase, rising from 0.31 million pounds in 1992 to 1.38 million pounds in 2016, with environmental persistence ranging from 47 to 120 days depending on the substance [129]. Patients infected with azole-resistant strains of *A. fumigatus* have mortality rates up to 33% higher than those with susceptible infections. Recent studies show a direct correlation between the intensity of agricultural triazole use and clinical resistance rates in the same geographic areas [130].

Ecological “hotspots” are areas where environmental conditions promote fungal growth in the presence of low azole concentrations, thereby favoring resistant strains. These hotspots include composting facilities that process agricultural waste with fungicide residues, urban areas with high fungicide use, and greenhouses with intense antifungal applications.

*Candida auris* illustrates the significant global threat from multi-drug-resistant fungi. Since its first identification in 2009, it has spread to all continents. Mortality rates range from 30% to 72%. In the United States, clinical cases increased during 2020–2021, with a threefold increase in echinocandin-resistant cases in 2021 alone, compounded by the strain on healthcare systems during the COVID-19 pandemic.

Recent surveillance data show worrying trends in antifungal resistance, with *C. auris* documented to be resistant to all drugs across four major categories of antifungals [131]. A major surveillance study of 190 wastewater treatment plants in the United States found *C. auris* nucleic acids in 19% of the facilities, indicating widespread environmental presence and possible community spread [132]. Data from New York and New Jersey (2016–2020) showed rising rates of fluconazole resistance (>90% of isolates) and emerging echinocandin resistance (15–20% of isolates) in clinical populations.

Effective antifungal management requires coordination across all sectors that use these drugs. This involves developing sector-specific guidelines that balance treatment efficacy with resistance prevention, investing in non-chemical control methods in agriculture, and implementing comprehensive resistance monitoring across all sectors. Successful examples of collaborative antifungal stewardship programs have emerged in Latin America, where joint efforts between human and veterinary medicine have resulted in significant reductions in antifungal resistance rates [133]. The introduction of One Health management frameworks has demonstrated measurable improvements in appropriate antifungal prescribing practices across health systems [134]. These programs highlight the crucial need to integrate antifungal stewardship principles across human, animal, and environmental health sectors to maintain the effectiveness of existing antifungal treatments.

## 4. Parasites

### 4.1. Parasitic Infections

Parasitic diseases caused by intestinal helminths and protozoa represent a global public health condition, individuals infected suffer from significant morbidity and mortality [135,136]. Approximately 3.5 billion people (30% of the world’s population) are affected, and 450 million patients suffer from parasitic infections [136], the majority of whom are children [137]. These infections represent a serious health problem, since they can cause iron deficiency, anemia, growth retardation in children, and other physical and mental health problems [137].

According to the World Health Organization (WHO), the most common intestinal parasitic infections are the soil-transmitted helminths, *Ascaris lumbricoides*, *Trichuris trichiura*, *Ancylostoma duodenale*, and *Necator americanus* [135]. The prevalence of intestinal parasitic infections (IPI) varies among countries considering geographical, social, and environmental factors [135]; this is also related to the mechanisms by which these diseases spread such as water runoff, rain, animals, and human migration amidst inadequate public sanitation and infrastructure [138]. However, helminths have been reported to cause gastrointestinal infections more frequently than protozoal infections in developing countries [136] where they are the most prevalent diseases, particularly in sub-Saharan Africa (SSA), Asia, Latin America and the Caribbean [135]. Regarding the intestinal protozoan parasites, *Giardia lamblia*, *Cryptosporidium parvum*, *Blastocystis* spp., *Cyclospora cayetanensis*, *Cystoisospora belli*, and *Entamoeba histolytica* are the most common in the United States [135].

In parasitology, the One Health approach focuses on parasitic zoonoses, vectors and vector-borne diseases, emerging infectious diseases, disease transmission at the wildlife/domestic animal/human interfaces, global health, climate change, food safety, and AMR [139]. Moreover, parasitic diseases associated with high morbidity or inadequate treatment options, and a variety of potential transmission routes, should also remain the focus of attention under the One Health umbrella [140].

### 4.2. Parasitic Zoonoses

With the rise of One Health as a public health discipline zoonotic parasitic infections represent a great interest, considering the shifting interactions between humans and other animals as well as global trade and agriculture [138]. In fact, most of the classic parasitic diseases due to protozoa, cestodes, trematodes, nematodes, pentastomids, or arthropods are zoonotic [137].

Zoonoses is an infection in humans caused by parasites of animals [141]. To date, more than 200 parasite species and the diseases they cause are of zoonotic origin and some of these diseases are transmitted to humans by their pets [137]. Parasites are not only the causative agents of animal and human diseases but are also often the main route for acquiring the infection [137]. Spill-over events from animals have been the catalyst for many human parasitic infections, several infections move between livestock and wildlife, evolving as they go with novel strains posing a major threat to new host populations who have not experienced these infections previously [142].

Toxoplasmosis is a parasitic infection that is often transmitted between domestic cats and their owners. This infection has been associated with miscarriage and psychiatric disorders and has been considered as the cause of severe pathogenesis in immunocompromised individuals [142].

Another example is visceral leishmaniasis, caused by *Leishmania donovani* and *Leishmania infantum*. Visceral leishmaniasis, is the most serious form of the disease and it is fatal if untreated. This disease can be transmitted from dogs to humans and is endemic in more than 65 countries, mostly distributed in Brazil, India, Ethiopia, Kenya, Somalia, South Sudan, and Sudan. The parasites are transmitted between mammalian hosts by more than 90 female phlebotomine sandfly species [137,143].

Chagas disease is a zoonosis with a particular threat to humans. Surveys of opossums and raccoons in Florida have shown infection rates with *T. cruzi* of approximately 50% as well as in peridomestic animals. Even more alarming, a citizen science project aimed at assessing the urban public health risk of Chagas disease in Caracas revealed *T. cruzi* infection rates of 80% in triatomines caught in domestic premises and a high proportion of human-positive bloodmeal [137]

Other examples are *S. mekongi* and *S. japonicum*; they are able to live in animal reservoirs including agricultural/domestic animals such as dogs, cats, pigs, goats, and horses. The prevalence of Schistosomiasis has increased due to the growing reliance on local agricultural produce and lack of adequate infrastructure and sanitation [138]

Zoonotic infections are increasingly being recognized, likely due to factors such as overpopulation, mass population migrations due to natural or human-made disasters, population migration to large urban centers, inadequate food and water supplies for humans, and settlements in areas where animal populations and parasites were previously isolated from humans. These phenomena cause widespread infections in domestic animals and humans, as well as bringing human hosts into contact with additional zoonotic infections. Therefore, changes in the epidemiology of zoonotic parasitic infections can be expected, with their emergence becoming increasingly important [144].

*Plasmodium knowlesi* is a good example of an emerging zoonosis. Recently identified in humans in 2000, it spreads from Macaques to humans via a mosquito vector. There has not yet been any evidence of human-to-human transmission, but due to the presence of the disease in Southeast Asia it may eventually occur [142].

Interactions between wildlife and human populations promote the emergence of zoonoses. Red fox populations have increased in urban centers due to rabies control in these animals which increases the exposure to Toxocara canis and other parasites [144].

Babeiosis is an Ixodid tick-borne zoonotic infectious disease caused by *Babesia* spp. To date, there are 100 *Babesia* spp. that have been recognized, infecting many mammalian and some avian species. *Babesia microti* complex, *Babesia divergens*, *Babesia bovis*, *Babesia canis*, *Babesia duncani,* and *Babesia enatorum* are those associated with human disease. The expansion of urban environments into undeveloped areas has increased the frequency of babesiosis and the recognition of new species that can infect humans [144].

Vector-borne diseases, which include viral, bacterial, or parasitic diseases that are transmitted by ectoparasites like ticks or mosquitoes, account for more than 17% of all human infectious diseases worldwide, causing more than 700,000 deaths annually [137].

One of the most known vector-borne parasitic diseases is malaria [137]. Malaria remains a major public health problem in most of the tropical world. More than 200 million cases and 435,000 deaths were estimated worldwide in 2017. Importantly, 11 countries contribute approximately 70% of estimated malaria cases and deaths globally. From these, 10 belong to sub-Saharan Africa and India. *Plasmodium falciparum* and *Plasmodium vivax* are responsible for the largest number and for the most severe cases of the disease [143].

Human behavior plays an important role in the epidemiology of parasitic diseases. Changes in demography and environmental alteration, climate change, technology, land use, etc., favor the emergence and spread of parasitic diseases [141]. Therefore, understanding the changing ecology and epidemiology of zoonotic parasitic infections and defining modes of transmission can lead to effective strategies to mitigate the further spread of infections [142].

### 4.3. Foodborne Parasitic Diseases

Foodborne parasitic diseases are produced by the ingestion of food contaminated with parasites. They represent significant public health problems due to their high incidence; according to the WHO, approximately 48% of parasitic diseases are food-borne. These diseases produce serious sequelae and mortality, as well as negative economic effects attributable to healthcare costs [145].

The frequency of foodborne parasitic diseases has increased due to several factors like a higher consumption of raw or slightly cooked vegetables, climate change, the increase in human population and urbanization, usage of contaminated or wastewater in food industry, as well as cultural preferences and changes in eating habits [145]

These diseases are mainly caused by trematodes; there are currently around 56 million people infected with foodborne trematodes [146]. *Taenia solium* is considered to be the number one food-borne parasite on a global scale [147]. Humans consume undercooked pork meat infected with the cysticerci (larval stage), which then develop to adult worms in the gut [146]. Humans may also become infected by directly consuming the eggs, either through the environment or autoinfection. This results in cysticercosis, an infection that affects at least 50 million people round the world. Cysts in the brain may produce headaches, seizures, and it can even be considered as a cause of epilepsy.

The increase in its incidence may be due to pigs being free roaming, meat inspection either being non-existent or insufficient, open defecation being highly prevalent, personal hygiene and meat hygiene being poor and the demand of pork is increasing [146,147].

Some of the more important parasites that cause food-borne disease worldwide are *Echinococcus granulosus*, *Toxoplasma gondii*, *Cryptosporidium* spp., *Entamoeba histolytica*, *Trichinchiella spiralis*, *Trypanosoma cruzi* and *Fasciola hepatica* [145]. Table 2 describes the food borne transmission pathways for the parasites mentioned above.

### 4.4. Effect of the Environment in Parasitic Infections

Wildlife parasitology recognizes the effects of environmental change on host–parasite relationships, a vital component in One health [139]. Parasites, as natural parts of ecosystems, can serve as indicators of high biodiversity and intact trophic relationships in healthy ecosystem [139]. In aquatic ecosystems they are important and integral elements that drive fundamental ecological processes, such as contributing to a system’s biodiversity, productivity and ecosystem engineering [151].

Enviromental parasitology may also have a medical approach, especially when the contamination and occurrence of infective parasitic stages in the environment is addressed [151,152]. The interactions between parasites and pollutants can affect the health of the host in different ways according to the type of pollutant, exposure concentration, and duration. Some pollutants suppress the immune response leading to higher parasite infection intensities. On the other hand, parasites themselves may also change the physiological or biochemical response of the host to a pollutant in different directions as both stressors might interact in a synergistically, antagonistically or additive way [151].

Environmental factors (both abiotic and biotic) can lead to context-dependent parasitism and virulence. Abiotic factors clearly affect the life cycles of the host and the parasite and, therefore, the epidemiology of outbreaks. Among these factors, one of the most important is climate change [153]. The relationship between climate change and parasitic infections is mediated to a large extent by changes in temperature, humidity, rainfall patterns, and extreme weather events. These changes produce great effects on the environment, enabling the spread of parasitic diseases previously confined to certain regions that were previously thought to be safe at risk [141,154]. Warming temperatures extend the range of tropical diseases such as malaria, fluctuating rainfall patterns contribute to the spread of water-borne and food-borne diseases, increased flooding and droughts lead to water contamination thus promoting parasite survival, and environmental disruptions impact the habitat of vectors and hosts, influencing parasite spread [154].

Global warming is changing some ecosystems faster than the capability of adaptation leading to possible extinction of a huge amount of species, including parasites [152]. Parasites are natural parts of ecosystems; therefore the understanding of the ecological dynamics of a parasite shared among species will be helpful for assessing and managing risks to one of the species [139].

Changes in the environment besides climate change, like alterations in the type of soil and its degree of water absorption, changes in vegetation characteristics, fluctuations in water bodies (size, shape, temperature, and pH), affect the ecological balance and context with vectors modifying their biodiversity, abundance, and behavior [155].

All this in conjunction has increased the risk for vector-borne diseases; an example is the spreading of triatomine bugs in the Americas that have established populations as far as Nebraska, and autochthonous populations of sandflies (*Phlebotomus mascittii*) have been reported from Vienna and Budapest [137].

Many parasites use the physical environment to transmit developmental stages of their life cycles, and most of them are transmitted through water, food and soil. Parasites are excreted in feces or urine into our environment, which are then either ingested by, or actively penetrate tissues of, a susceptible host [156]. The use of untreated feces as fertilizer, the use of untreated sewage effluent for irrigation, and defecation by agricultural workers in or near the fields where they work leads to contamination of soil, food, and water [156].

Food borne parasitic outbreaks have been produced by using contaminated water from sewage, waste-water effluent, and muck spreading, among others. Food normally becomes a potential source of human infection by contamination during production, collection, transport, preparation or processing. The sources of such contamination are usually feces, fecal contaminated soil or water or infected food handlers [156].

Contaminated potable water from community water systems is especially important as it can deliver parasites to numerous consumers, many of whom become infected. Over 160 water-borne outbreaks of giardiasis and cryptosporidiosis, affecting more than 450,000 individuals have been reported. These parasites are considered significant water-borne pathogens in the developed world because they are small enough to penetrate water treatment processes, are insensitive to the disinfectants commonly used in water treatment and, due to densities of environmental contamination with infective cysts and oocysts are sufficient to pollute the aquatic environment [156].

Soil and herbage are important vehicles for transmitting ova, larvae, cysts and oocysts, with both person-to-person and zoonotic transmission being documented. *A. lumbricoides*, *T. trichiura*, *Toxocara* spp., *Fasciola* spp., *Trichinella* spp., *Toxoplasma gondii*, *Echinococcus* spp., *Opisthorchis* spp., *Clonorchis* spp., *Taenia solium*, and *Strongyloides* spp. can be significant contaminants for these environments [146,156].

The potential for environmental contamination depends upon different factors including the number of infected hosts, the number of transmissive stages excreted, human activity, socio-economic and ethnic differences in behavior, geographic distribution, sanitation, and safety of drinking water sources [156].

### 4.5. Antiparasitic Drug Resistance

The noteworthy impact of human protozoan infections has been augmented by the lack of effective vaccines and safe and affordable drugs [143]. Moreover, the efficiency of the available drugs is being reduced by the development of parasite drug resistance. This phenomenon has been described in both helminths and protozoan parasites [15,143].

Drug resistance of *Plasmodium*, primarily *Plasmodium falciparum* and *Plasmodium vivax,* is one of the reasons why antimalarial drugs failed and for the stalling progress in malaria elimination [15,157]. Resistance was first observed against Quinine in 1910 and afterwards against Proguanil in 1948 [133]. Since then, resistance against Pyrimethamine, Mepacrine, Sulfadoxin-pyrimethamine, Mefloquine and Chloroquine has been described in different regions such as Africa, Southeast Asia, Thailand, Cambodia, Colombia, Indonesia and Nueva Guinea [158].

At present, resistance has been described for almost all available drugs [157]. Chloroquine is the first-line treatment for *P. vivax* in endemic countries however it faces resistance in different parts of the world, mainly in regions with a high prevalence of *Plasmodium vivax* like Indonesia and Oceania, considered epicenters of chloroquine resistance [159].

Recent evidence shows that parasites are becoming resistant to the newest agents [157]. One of the major problems that has contributed to resistance is the widespread and indiscriminate use of drugs, contributing to evolving mechanisms of resistance, such as a higher genetic mutation rate and gene amplifications [159]. Other important factors include the strength of the drug selected, the treatment compliance, poor adherence to treatment, improper dosing, and the use of drugs with poor pharmacokinetic properties [159]. It is important to mention that the used of falsified antimalarial drugs without active pharmaceutical ingredients (e.g., halofantrine instead of artemisinin) also contribute to resistance, producing as a side-effect of hypergametocyopaenia [159].

Giardiasis, a globally important parasitic infection, is associated with increasing rates of drug resistance and treatment failures [15]. The infection caused by *Giardia duodenalis* is treated mainly by the use of albendazole and metronidazole; however therapeutic failures have been observed probably due to the induction of drug resistance [15,160]. One of the possible reason for this resistance is that in some countries a suboptimal dose has been used in community-based treatment programs for de-worming children [160]

In the field of helminthology, drug resistance is observed almost exclusively in animals. In countries like Australia or some located in South Africa and South America anthelmintic resistance (AR) is an important disease problem; it observed with frequencies exceeding 50% in sheep, goats and horses [161]. The problem is so severe that in Paraguay, a resistance to all available broad-spectrum anthelmintics is observed, forcing farmers to give up raising sheep due to the undefeatable problems with anthelmintic resistance [161].

In contrast, there is scarce information on anthelmintic resistance in humans. To date, there are reports of AR only among nematodes and trematodes but not in cestodes [161]. In the case of nematodes, it has been described a failure of mebendazole in treating *Necator Americanus* in Mali, and poor efficacy of pyrantel pamoate against *A. duodenale* in northwestern Australia [161].

Albendazole and mebendazole have a broad-spectrum activity against intestinal nematodes. The inappropriate use of these drugs led to a decreased efficacy linked to the selection of single nucleotide polymorphisms of the gene that codifies for the β-tubulin. Studies have correlated resistance to both albendazole and mebendazole with a conserved mutation (p.Phe200Tyr) in β-tubulin isotype 1 (p.Phe200Tyr) [161]. Thus, the excessive use of these drugs should be addressed to avoid the increasing rates of resistance.

On the other hand, drug resistance in trematodes is frequently documented. Resistance of schistosomes to oxamniquine is reported and is limited to scattered areas in Brazil. Praziquantel has replaced oxaminiquine in different regions, unfortunately recent reports on the possible development of resistance to Praziquantel have generated great concern since it is one of the current control strategies to reduced morbidity in populations affected [161].

As with other drugs, the risk of resistance in anthelmintics increases during underdosing as well as with frequent applications of the same class of anthelmintics. A single drug, which is usually very effective in the first years, is often used continuously until it no longer works. For this reason, one of the strategies to prevent the development of resistance is to rotate drugs and avoid long-term use [161].

There is also strong evidence that resistance develops more rapidly in regions where animals are dewormed regularly [161]. Correct dosing and administration are very important, as studies have shown that underdosing contribute to the selection of resistant or tolerant strains. The use of poor-quality drugs (generic products of substandard quality, repacked and/or reformulated products, and expired drugs) in human and veterinary medicine may also contribute to antiparasitic drug resistance. Prophylactic strategies include a reliable diagnosis to determine the type of worm and avoiding of profilacting mass treatments mainly in domestic animals [161,162].

One of the main challenges is the insufficient tools and methods available to detect AR problems on time. Also, the laboratory confirmation is still compromised by the lack of standardization methods and reference material; this is why implementation of protocols and collection of data is necessary [161].

## 5. Viruses

### 5.1. Viral Zoonoses

Viral zoonoses represent a growing challenge to global public health, driven by the intensification of human–animal interfaces and increasing anthropogenic pressure on natural ecosystems. Factors such as deforestation, loss of biodiversity, the illegal wildlife trade, and intensive livestock production have reduced the natural barriers between humans and animals, increasing opportunities for pathogen contact and transmission [163,164].

**Figure 3:**
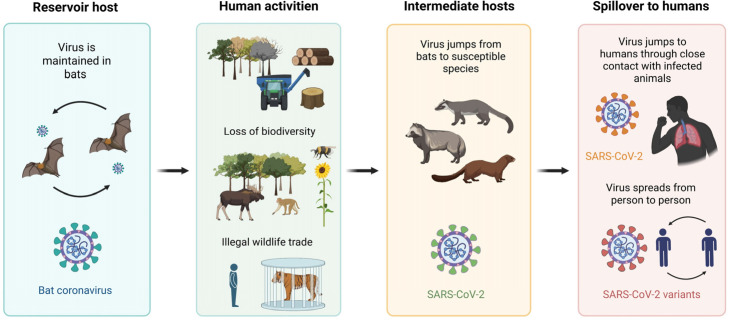
Viral zoonoses transmission, example of SARS-CoV-2.

Over the last few decades, zoonotic outbreaks and pandemics have demonstrated the magnitude of this risk. The most recent is the SARS-CoV-2 pandemic, with not only bats and pangolins as potential reservoirs, but predominant person-to-person transmission being shown as well. (Figure 3) [165]. Other examples include severe acute respiratory syndrome (SARS, in 2022), which originated in civets and bats of the genus *Rhinolophus* [166]; Middle East respiratory syndrome (MERS, in 2012), associated with animal reservoirs in the Arabian Peninsula [167]; Ebola virus disease in Africa; Nipah virus outbreaks linked to Pteropus bats [168,169]; and the 2009 influenza A (H1N1) pandemic, which involved swine as an intermediate host [170].

Wildlife markets constitute high-risk environments for the emergence of infectious diseases by bringing together multiple species of different origins under unsanitary and high-density conditions [171]. This favors interspecies mixing, pathogen transmission, and recombination, as well as immunological stress in the animals, increasing virulence and dissemination capacity [172]. Species such as bats and rodents stand out as natural reservoirs for various viruses, including coronaviruses, henipaviruses, filoviruses, hantaviruses, and mamarenaviruses [173].

Coronaviruses (CoVs) in particular, illustrate the pandemic potential of these pathogens. Of more than 300 identified CoVs, 7 are known to infect humans, and of these, SARS-CoV, MERS-CoV, and SARS-CoV-2 have caused epidemics and pandemics with significant health impacts [174]. The genetic diversity and recombination capacity of these viruses, coupled with their circulation in multiple animal reservoirs, make it difficult to predict their potential [175]. SARS-CoV-2 is a prime example of this genetic diversity and recombination ability, having demonstrated a great capacity for adaptation, immune evasion, and alteration of epidemiological dynamics, leading to the emergence of variants such as Alpha, Delta, and Omicron [176].

### 5.2. Effect of the Environment in Viral Infections

Viruses, recognized as the most abundant and diverse biological entities on Earth, have traditionally been studied in the context of infectious diseases affecting humans, animals, and plants. However, they transcend their conventional perception as mere pathogenic agents [177,178]. Structurally simple comprising a protein capsid that encloses their genetic material (either DNA or RNA), and in some cases, a lipid envelope derived from the host cell, they lack metabolic autonomy [178]. Nonetheless, their ability to process genetic information enables them to fulfill essential ecological roles. In marine environments, where they are particularly abundant, viruses regulate microbial populations, facilitate nutrient recycling, and contribute to ecological homeostasis. Moreover, their influence on human evolution is profound, as it is estimated that viral sequences constitute approximately eight percent of the human genome [179].

Pathogenic viruses, including emerging and re-emerging pathogens such as human coronaviruses, are primarily transmitted via aerosols (particles < 5 µm), droplets (particles > 5 µm), fomites, and direct contact. These transmission mechanisms are particularly significant in environments characterized by high population density and inadequate ventilation, such as hospitals, hotels, and shared human living spaces. Understanding these transmission pathways is essential for the implementation of effective preventive measures, including surface disinfection, proper ventilation, air filtration, humidity control, and environmental temperature regulation [180,181,182,183].

To monitor viral spread, wastewater-based epidemiology (WBE) has emerged as an innovative and effective tool, as demonstrated during the SARS-CoV-2 pandemic, where the detection of viral RNA in wastewater showed a direct correlation with increases in clinical cases and hospitalizations [184]. Likewise, during the COVID-19 pandemic, wastewater surveillance provided critical information regarding the infection burden, including asymptomatic cases and the circulation of SARS-CoV-2 variants, thereby modernizing outbreak response strategies [185].

This technique not only enables the identification of pathogens through molecular biology methods for the extraction and analysis of genetic material (DNA or RNA, depending on the agent), but also provides continuous and timely data to inform public health decision- making [186]. The versatility of wastewater-based epidemiology (WBE) has allowed its application to extend beyond SARS-CoV-2. For instance, the analysis of human adenovirus (HAdV-F40 and HAdV-F41) DNA concentrations in wastewater during acute hepatitis outbreaks, combined with genomic sequencing, has enabled comparisons between environmental and clinical viral variants, confirming that this approach can complement traditional surveillance systems [187,188]. WBE has also been used to detect monkeypox virus (Mpox), whose excretion in feces and urine allows for its identification in wastewater to track non-hospitalized cases and monitor transmission trends [189]. Recently, in the United States, this approach has even been employed to monitor avian influenza A (H5N1) in human populations, demonstrating its potential as a comprehensive early warning system for various emerging pathogens [190]. Thus, wastewater-based epidemiology represents a transformative advancement in infectious disease surveillance by offering timely and holistic data which, when integrated with traditional public health systems, supports more effective management of current and future outbreaks (Table 3).

### 5.3. Antiviral Resistance

Antiviral resistance represents one of the greatest challenges in the control of viral diseases, as the adaptive capacity of viruses often outpaces the development of new therapeutic agents [191,192].

Direct-acting antivirals (DAAs) are currently the most widely used due to their high efficacy and specificity. These agents function by targeting and inhibiting essential viral proteins to block replication. However, they face a critical limitation: a low genetic barrier to resistance. This vulnerability allows viruses to rapidly acquire mutations through evolutionary selection, thereby reducing drug susceptibility and compromising therapeutic efficacy [193].

Resistance mechanisms are diverse but share a common foundation: the emergence of genetic mutations that enable viruses to evade the action of antiviral drugs. The human immunodeficiency virus (HIV) is a pleomorphic retrovirus characterized by a positive sense single-stranded RNA (ssRNA+) genome. During replication, this RNA genome is reverse transcribed into double-stranded DNA (dsDNA) by the viral enzyme reverse transcriptase. Nucleoside reverse transcriptase inhibitors (NRTIs), such as lamivudine, exert their effect by disrupting the geometry of the enzyme’s catalytic site, particularly within a highly conserved region that includes the M184 motif. As a result, mutations such as M184V in reverse transcriptase confer resistance to lamivudine and related drugs [194].

The influenza virus is an enveloped pathogen with spherical or pleomorphic morphology and a segmented genome composed of eight negative-sense, single-stranded RNA (ssRNA−) molecules. These segments encode several viral proteins, including the subunits of the heterotrimeric RNA-dependent RNA polymerase (RdRp) complex (PA, PB1, and PB2), which mediates genome replication and transcription in the host cell nucleus. A key function of this polymerase is the cap-dependent endonuclease activity located in the PA subunit, essential for initiating viral mRNA synthesis through the cap-snatching mechanism. The antiviral drug baloxavir targets this highly conserved endonuclease domain, thereby blocking cap-snatching, disrupting viral replication, and providing broad-spectrum activity against influenza A, B, C, and D viruses. However, resistance-associated mutations, such as the PA-I38T substitution, diminish the binding affinity of baloxavir for its enzymatic target and substantially reduce its therapeutic efficacy [195,196].

In SARS-CoV-2, the dimeric cysteine protease (3CL) is essential for viral replication, as it cleaves the viral polypeptides 1a and 1ab to release the non-structural proteins (nsps) required for the viral life cycle. Nirmatrelvir inhibits 3CL, preventing the liberation of functional nsps and thus halting replication. Nevertheless, nirmatrelvir’s potency can be compromised by mutations in the 3CL active site. The E166V mutation eliminates key hydrogen bonds required for drug binding to the target site, reducing inhibitory potency by more than tenfold [197].

Similarly, in hepatitis C virus (HCV), the Y93H mutation in the NS5A protein can reduce the efficacy of daclatasvir by up to 1000-fold. This occurs because daclatasvir is a direct-acting antiviral designed to target the NS5A protein, a non-structural viral phosphoprotein that is an essential component of the functional replication complex. This complex, located on the membranes of the endoplasmic reticulum, is responsible for amplifying the viral RNA genome. Daclatasvir works by specifically inhibiting the critical functions of NS5A, thereby interrupting HCV replication and causing a decrease in serum HCV RNA levels. The Y93H mutation directly alters this target, severely compromising the drug’s ability to bind and exert its antiviral effect [198,199]. These resistance processes are accelerated by factors such as monotherapy, irregular adherence to treatment, and the transmission of resistant strains—an especially concerning issues in regions with fragile healthcare systems [200].

Antiviral resistance constitutes a complex threat that demands multidisciplinary responses; the integration of combination therapies, genomic surveillance, and the development of high-genetic barrier antivirals will be essential to preserve therapeutic efficacy.

## 6. Conclusions

The One Health approach for infectious diseases needs ongoing investment in research and development in several areas. This includes creating new antimicrobials agents with unique ways to work, funding research on vaccines, developing quick and affordable diagnostic tools for use in human, animal, and environmental health, and improving our understanding of how these diseases spread and the risk factors involved at One Health interfaces.

Effective implementation requires integrating policies across government sectors. This means aligning regulatory frameworks for antimicrobials use, building intersectoral surveillance systems, and coordinating responses during outbreaks. Building capability involves investing in education and training across different fields, particularly in One Health principles and recognizing zoonotic diseases.

New technologies provide fresh opportunities for One Health surveillance and response. This includes genomic surveillance for tracking transmission and resistance in real time, environmental monitoring using molecular tools to detect different microorganisms, predictive modeling that combines climate and epidemiological data, and digital health applications to improve surveillance and case management.

## Figures and Tables

**Table 1:** Antibiotic resistance trends in humans, animals, and the environment (One Health perspective).

Biological System	Setting/Pathogen	Period/Region	Metric/Trend	Implication	Reference
Human (global)	GLASS indicators (*E. coli* 3GC-R; MRSA)	WHO GLASS data (2023)	Global median: 42% *E. coli* resistant to 3rd-gen cephalosporins; 35% MRSA	High pressure on carbapenems; urgent need for stewardship and surveillance	WHO 2023 [97]
Human (EU/EEA)	*K. pneumoniae* bloodstream infections/carbapenems	EARS-Net 2019–2023	Incidence of 57.5% since 2019 for carbapenem-resistant *K. pneumoniae*	Rising trend in Gram-negatives; strengthen hospital infection control	ECDC 2023 [98]
Human (Latin America)	ICU: *A. baumannii* and *P. aeruginosa*/carbapenems	Regional review (2005–2015)	*A. baumannii* CR often >50%; *P. aeruginosa* CR 20–60%	Need to optimize β-lactam use and improve infection control in ICUs	Fabre et al., 2022 [99]
Human (hospital environment)	Sinks/drains as reservoirs; *K. pneumoniae* NDM	2017–2025 (multiple reports)	NDM outbreaks linked to sink traps and drains	Plumbing redesign and biofilm-targeted cleaning are critical	Kotay et al., 2017 [100]; Bourigault et al., 2025 [101]; McCallum et al., 2025 [102]
Poultry	Ceftiofur metaphylaxis—*Salmonella* Heidelberg 3GC-R	Canada 2003–2008	Resistance rose from 5% to 35%; withdrawal reduced to 7%	Ban non-therapeutic use; respect withdrawal periods	Dutil et al., 2010 [82]
Young cattle	Florfenicol sub-therapeutic dosing—gut resistome	USA 2024	Shifts in resistome and microbiota profiles	Avoid sub-therapeutic dosing; monitor resistome changes	Berge et al., 2005 [103]
Animal–environment (vectors)	Ticks from livestock—ARGs (ESBL, aminoglycosides, etc.)	Saudi Arabia 2023	ARGs detected in camel tick microbiomes	Arthropods are overlooked AMR conduits	Aljasham et al., 2023 [104]
Environment (wastewater)	AOPs (ozone, UV/ozone) against ARGs	Europe 2018	Ozone: 85–98% ARG reduction; UV + O3: 84–99%	Advanced AOPs effective; dose and by-product risks need management	Jäger et al., 2018 [96]
	Ozonation/UV254 nm—effectiveness and risks	Portugal 2017	Substantial ARG removal; risk of transient selection	Optimize exposure times and combine with biofiltration	Sousa et al., 2017 [105]
Environment–urban	Urban sewage—global resistome	Global 2019	Resistome reflects human antimicrobial use	Wastewater metagenomics as a surveillance tool	Hendriksen et al., 2019 [73]
	MetaSUB project—urban microbiomes	60 cities, 2015–2017	AMR markers vary by city and climate	Spatial mapping for targeted interventions	Danko et al., 2021 [74]

AMR—Antimicrobial resistance; ARGs—Antimicrobial resistance genes; ARB—Antibiotic-resistant bacteria; AOPs—Advanced oxidation processes; 3GC-R—Third-generation cephalosporin resistant; MRSA—Methicillin-resistant *Staphylococcus aureus*; EARS-Net—European Antimicrobial Resistance Surveillance Network; ECDC—European Centre for Disease Prevention and Control; GLASS—Global Antimicrobial Resistance and Use Surveillance System; NDM—New Delhi metallo-β-lactamase; CR—Carbapenem-resistant; ICU—Intensive Care Unit; WWTP—Wastewater Treatment Plant; MBR—Membrane Bioreactor; UF—Ultrafiltration; SXT—Trimethoprim–sulfamethoxazole; ESBL—Extended-spectrum β-lactamase.

**Table 2:** Foodborne parasites transmission pathways [148,149,150].

Parasite	Parasite
*Cryptosporidium* spp.	Fruit and vegetables: contaminated with feces of animals, contaminated cultivation water, infected handlers during production process, contaminated wash water during packaging and sale Fruit and vegetable juice: contaminated with feces of animals, contaminated water used for dilution, infected handlers during production process Dairy products: contaminated with feces of infected animals during milking, infected handlers during production process Molluscan shellfish: contaminated by seawater during growing, infected handlers during production process Meat: contaminated by feces/intestinal content of infected animals at the abattoir during slaughter, infected handlers, infected surfaces
*Toxoplasma gondii*	Fruit, vegetables and herbs: contaminated with feces of animals, contaminated cultivation water Fruit and vegetable juice: contaminated with feces of infected felids during cultivation of the crop Dairy products: contaminated by the transfer of tachyzoites to milk of lactating infected mammals such as goats Molluscan shellfish: contaminated by seawater during growing, cross contamination during depuration Meat: that is not adequately treated prior to consumption, infected surfaces, infected handlers
*Echinococcus* spp.	Fruit, vegetables and herbs: contaminated with feces of dogs, foxes and other canids during cultivation, contaminated water used for irrigation Fruit and vegetable juice: contaminated with feces of infected felids during cultivation of the crop, contaminated water used for dilution Drinking water
*Giardia lamblia*	Fruits and vegetables: contaminated with feces of animals and humans, contaminated cultivation water Molluscan shellfish: contaminated by seawater during growing Drinking water
*Fasciola hepatica*	Aquatic plants: such as watercress contaminated with feces, contaminated cultivation water Meat: that is not adequately treated prior to consumption, infected handlers, infected surfaces Drinking water
*Trypanosoma cruzi*	Fruit and vegetable juice: contaminated with feces from infected bugs.
*Paragonimus westermani*	Snails and crustacean: infected with metacercariae
*Diphyllobothrium latum*	Salmonid and other freshwater/sea fish: infected with plerocercoid
*Taenia soilium*	Meat: (pig, camel, rabbit, bear) infected with cyst stages of the parasite, infected surfaces, infected handlers
*Taenia saginata*	Meat: (bovine and cervine) infected with cyst stages of the parasite, infected surfaces, infected handlers
*Cyclospora cayetanensis*	Fruit: specially raspberries contaminated with feces, contaminated cultivation water
*Trichinella* spp.	Meat: (pigs, bears, wild boar, warthog, walrus, seal) infected with cyst stages of the parasites, infected surfaces, infected handlers

**Table 3:** Tracking viruses via wastewater-based epidemiology.

Virus	Gene targets	Analysis Method	Location	Autor
SARS-CoV-2	*N1*, *N2*	RT-qPCR	Boston, MA, USA	Xiao et al. (2022) [184]
SARS-CoV-2	*N1*, *N2*	RT-ddPCR/RT-qPCR	Houston, TX, USA	Hopkins et al. (2023) [185]
HAdV-F41	*HAdV-F40/41*	RT-ddPCR/RT-qPCR	Northern Irish, UK	Reyne et al. (2023) [187]
SARS-CoV-2	*N1*, *Env*	RT-qPCR	São Tomé, São Tomé and Príncipe	Toancha et al. (2024) [188]
Hepatitis A	Target gene unspecified			
Enterovirus	*panEV*			
Poliovirus	*panPV*			
Mpox Virus (MPV)	Vi07922155_s1 (TaqMan)	qPCR	Poznan, Polonia	Gazecka et al. (2023) [189]
Influenza A Virus (IAV)	M-gene	dd-RT-PCR	Texas, North Carolina, and Hawaii, USA	Wolfe et al. (2024) [190]
Influenza A H5 Subtype	H5-gene (Hemagglutinin)			

SARS-CoV-2: Severe acute respiratory syndrome coronavirus 2, *Env*: Envelope gene, *HAdV-F40/41*: Human Adenovirus serotypes F40 and F41, HAV: Hepatitis A Virus, H5-gene: Hemagglutinin 5 gene, IAV: Influenza A virus, M-gene: Matrix gene, MPV: Mpox virus (formerly Monkeypox Virus), *N1*, *N2*: Nucleocapsid genes 1 and 2, *panEV*: pan-Enterovirus (assay targeting a conserved region), *panPV*: pan-Poliovirus (assay targeting a conserved region), qPCR: Quantitative Polymerase Chain Reaction, RT-qPCR: Reverse Transcription Quantitative Polymerase Chain Reaction, dd-RT-PCR: Droplet Digital Reverse Transcription Polymerase Chain Reaction.

## Data Availability

The original contributions presented in this study are included in the article. Further inquiries can be directed to the corresponding authors.
